# Does Otovestibular Loss in the Autosomal Dominant Disorder DFNA9 Have an Impact of on Cognition? A Systematic Review

**DOI:** 10.3389/fnins.2017.00735

**Published:** 2018-01-09

**Authors:** Jonas De Belder, Stijn Matthysen, Annes J. Claes, Griet Mertens, Paul Van de Heyning, Vincent Van Rompaey

**Affiliations:** ^1^Faculty of Medicine and Health Sciences, University of Antwerp, Antwerp, Belgium; ^2^Department of Otorhinolaryngology and Head and Neck Surgery, Antwerp University Hospital, Edegem, Belgium

**Keywords:** DFNA9, cognition, systematic review, quality of life, labyrinth diseases

## Abstract

**Background and Purpose:** Cognitive impairment has been observed in patients with bilateral vestibular loss (BVL) and in patients with sensorineural hearing loss (SNHL). DFNA9 is an autosomal dominant disorder that causes a combination of both sensory deficits by the 3rd to 5th decade. We therefore hypothesize a combined detrimental effect on cognition. The aim of this systematic review was to identify studies related to DFNA9 in general and its relationship with cognitive impairment more specifically.

**Materials and Methods:** Several databases including Medline, Cochrane Database of Systematic Reviews, Cochrane Central Register of Controlled Trials, ISI Web of Knowledge, and Web of Science were searched to accumulate information about DFNA9-mutations, including phenotype, genotype, pathophysiology, quality of life (QOL), and imaging in general and cognitive function more specifically. A qualitative analysis was performed on the 55 articles that qualified.

**Results:** The clinical features of DFNA9 are different along the 24 COCH mutations, described up to now. Vestibular symptoms generally present themselves a few years after SNHL onset in mutations associated with the vWFA-domain although they can precede SNHL onset in other mutations associated with the LCCL-domain. QoL has not been studied extensively in DFNA9, although scarce work is available on the positive impact of cochlear implantation to rehabilitate hearing. No studies were found evaluating cognition in DFNA9 patients.

**Conclusion:** Although cognitive impairment has been demonstrated in patients with hearing loss as well as in patients with BVL, no studies have been reported on the combination of both sensory deficits, such as in DFNA9. Further research is warranted to correlate otovestibular status with cognition.

## Introduction

Sensory input may be an important determinant between normal and pathological cognitive aging. Over the past few years, the relationship between hearing loss and cognitive impairment has been studied extensively in the aging population. Large prospective studies have found an independent relationship between hearing loss on the one hand and age-related cognitive decline and incident dementia on the other hand (Lin et al., 2011, 2013; Gallacher et al., 2012; Gurgel et al., 2014; Fulton et al., 2015; Castiglione et al., 2016; Wuyts et al., 2016). The mechanistic basis of this correlation remains unclear: hearing loss may accelerate cognitive decline in older adults and therefore acts as a risk factor of cognitive decline. Alternatively, hearing loss could be an early symptom of cognitive decline and could be an effect rather than a cause of cognitive impairment. A common cause that induces both pathologies may be a third underlying mechanism of the association (Martini et al., 2014; Peracino, 2014).

Cognitive deficits were also observed in animal and human studies on bilateral vestibular loss (BVL) (Smith et al., 2005). In individuals with BVL, increased vigilance is necessary to avoid falling, which makes multitasking difficult: e.g., patients need to stop walking in order to talk (Lundin-Olsson et al., 1997; Bessot et al., 2012). Furthermore, a reduction of gray matter volume was observed in BVL patients in the bilateral hippocampal region CA3 (Göttlich et al., 2016). Atrophy of the hippocampus in BVL patients resulted in emotional, navigational, spatial memory, and spatial anxiety deficits (Brandt et al., 2005; Fanselow and Dong, 2010; Kremmyda et al., 2016).

Hereditary hearing loss can be classified as syndromic hearing loss and non-syndromic hearing loss. DFNA9 is a cause of autosomal dominant non-syndromic late-onset sensorineural hearing loss (SNHL) associated with progressive BVL (Chen et al., 2013). It is caused by mutations in the COCH (coagulation factor C homology) gene, found on the long arm of chromosome 14 (14q12-q13). This gene encodes for the cochlin protein, which is highly expressed in the inner ear and found in lower levels in the spleen and very low levels in the eye, cerebellum and brain stem, kidney and liver (Bischoff et al., 2005; Li et al., 2005; Cho et al., 2012).

The function of cochlin is not fully understood though it is known to assist in structural support, sound processing, and maintenance of balance within the inner ear (Gallant et al., 2013). Animal models suggests an immune-mediating effect by regulating cytokine production, recruitment of immune effector cells, and bacterial clearance (Py et al., 2013). DFNA9 overexpression is also associated with raised intraocular pressure within patients and mice (Bischoff et al., 2007; Goel et al., 2012; Verbecque et al., 2017).

Although the pathophysiology behind the progressive bilateral cochleovestibular loss is not entirely understood, mutated cochlin deposits in the inner ear are considered pathognomonic (Robertson et al., 2006). Recent work has established the individual impact of hearing impairment as well as BVL on quality of life (QoL) and cognitive function (Smith et al., 2005; Vermeire et al., 2006; Guinand et al., 2012; Besnard et al., 2015; Popp et al., 2017).

Our objective was to perform a systematic review on DFNA9, specifically focusing on its phenotype, genotype, pathophysiology, histologic findings, imaging findings, QoL on the one hand and its effect on cognitive function on the other hand.

## Materials and methods

The strategy used and the reporting hereafter is based on the Meta-analysis of Observational Studies in Epidemiology statement (Stroup et al., 2000), and follows Cochrane guidelines (Green, 2014).

We focused on several bibliographical databases to identify relevant reports in English: Medline, Cochrane Database of Systematic Reviews, Cochrane Central Register of Controlled Trials, ISI Web of Knowledge, and Web of Science. The search was performed on 1 July 2016, and also included articles published ahead of print. The global search term was adapted to all databases (Green, 2014; Scholten et al., 2014). We searched for etiology, pathophysiology, QoL, MRI, CT, and cognitive function as outcome (O) of the studies. We included studies in English. Single case reports and systematic reviews were excluded.

The following search terms were combined for the PubMed / Medline search: (((((bilateral vestibulopathy) OR vestibular areflexia) OR DFNA9) OR Bilateral semicircular canal stenosis) OR semicircular canal fibrosis) AND ((((((Causality[Mesh]) OR Etiology[Subheading]) OR Pathophysiology[Subheading]) OR QoL) OR CT) OR MRI OR Cognitive function) For Web of Science, the following search terms were used: [TS = (bilateral vestibulopathy) OR TS = (vestibular areflexia) OR TS = (DFNA9) OR TS = (Bilateral semicircular canal stenosis) OR TS = (semicircular canal fibrosis)] AND [TS = (Causality) OR TS = (Etiology) OR TS = (Pathophysiology) OR TS = (QoL) OR TS = (CT) OR TS = (MRI) OR TS = Cognitive function].

**Figure 1:**
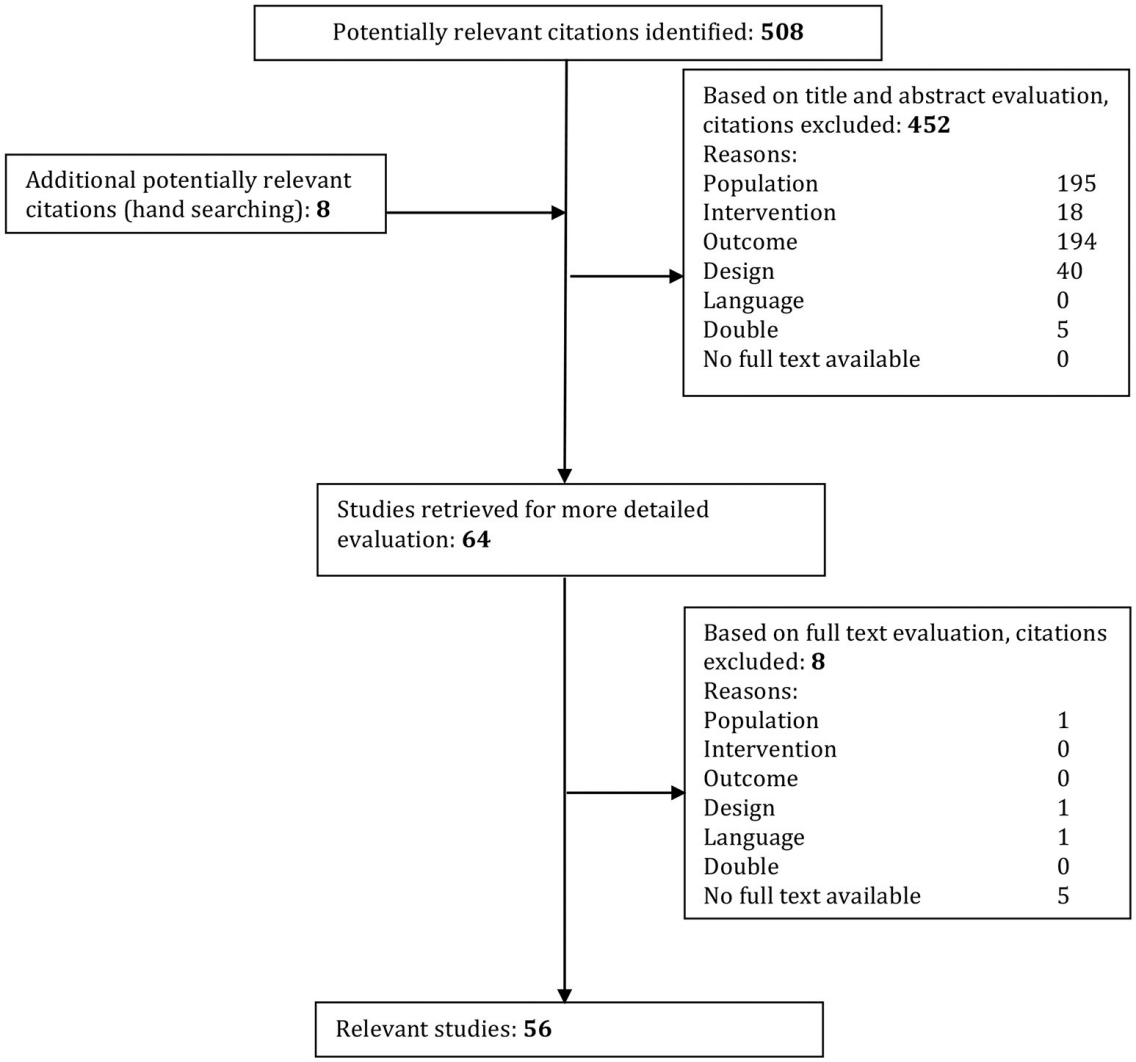
Systematic study inclusion. Studies were excluded if they conformed to the following criteria: Population: unilateral vestibulopathy, absence of DFNA9, pathology not concerning bilateral vestibulopathy or not cause by DFNA9 mutation. Intervention, not strictly observational; Outcome, When the goal of the study was not to acquire data involving the etiology or pathophysiology; Design, Reviews and single case studies; Language, Not English or Dutch. Double, If found in more than one database that article was only used once.

All studies were screened for eligibility in two screening phases based on the inclusion and exclusion criteria. In the first phase, all studies were screened on title and abstract by two reviewers (JDB, SM). If there was no abstract present but the title was applicable, the study was included to the second phase. In the second phase, the studies were screened in full-text using the same inclusion and exclusion criteria. The flow of included articles can be found in Figure 1.

The articles written by Dodson et al. (2012) and (Choi et al. (2013) were added based on references within other relevant articles. To get a better understanding of the function of cochlin within the eye we also included Goel et al. (2012).

## Results

### Phenotype

DFNA9 is characterized by progressive bilateral SNHL. The age of onset varies depending on the mutation although the average age of onset lies around the 3rd−5th decade (Chen et al., 2013). SNHL typically starts in the higher frequencies at the age of onset with an evolution toward deafness (Bom et al., 2003; Gao et al., 2013).

Dizziness is another important clinical aspect in most cases. Patients may initially complain of episodic vertigo spells and evolve toward BVL, which causes oscillopsia and imbalance (especially in the dark), difficulty while cycling etc. The vestibular symptoms generally present themselves a few years after the SNHL onset although there are some mutations where the vestibular symptoms present themselves simultaneously or even prior to SNHL onset (G88E, P51S, G87V, G87W; Bom et al., 1999a,b; Khetarpal, 2000; Bischoff et al., 2005; Kemperman et al., 2005; Collin et al., 2006; Chen et al., 2013). One exception was found, i.e., the P98H mutation, which presented with unilateral congenital hearing loss in a single case. (Dodson et al., 2012) The presentation of Ménière-like symptoms (tinnitus, vertigo spells, and hearing loss) is not uncommon. However, Ménière's disease typically presents with a more fluctuating pattern of hearing and vestibular symptoms, and a low-frequency SNHL. (Fransen et al., 1999; Wuyts et al., 2016) BVL symptoms may lead to reduced vitality, fear of falling and reduced general health, further described in the “QoL” paragraph. One study has also documented the absence of cervical vestibular-evoked myogenic potentials (cVEMPs), indicating saccular otolithic deficits (Robertson et al., 2008).

### Genotype

The COCH gene is located on chromosome 14q12-13 which encodes for cochlin (Manolis et al., 1996). We identified 24 different mutations in the COCH gene, autosomal dominantly inherited and heterozygous (Table 1). COCH encodes for a 550-amino-acid protein called cochlin. The COCH gene contains the following domains: an N-terminal signal peptide (SP), a late gestation lung protein Lgl1 (LCCL) domain, two vWFA domains (von Willebrand factor A-like) and two short intervening domains (ivd) (Robertson et al., 2001; Gallant et al., 2013; Bae et al., 2014). The vestibular symptoms are more correlated with mutations within the LCCL domain than to mutations within the vWFA domains. Individuals with mutations within the vWFA domain usual have an earlier onset of hearing loss than those with mutations in the LCCL domain (Bae et al., 2014).

**Table 1:** Known COCH mutations and clinical features.

**Effected protein**	**Domain**	**Progressive hearing loss**	**Decade of onset**	**Vestibular involvement**	**Accompanied by tinnitus**	**Ethnicity**	**References**
p.A119T	LCCL	Yes	4th	Present in all	–	Japanese	Pauw et al., 2011
p.A487P	vWFA2	Yes	2^nd^	Present in some	–	Italian	Bae et al., 2014
p.C162Y	Intervening	Yes	2nd	Not present	–	Chinese	Gao et al., 2013
p.C542F	vWFA2	Yes	2nd−5th	Present in some	–	USA	Street et al., 2005; Yuan et al., 2008
p.C542R	vWFA2	Yes	2nd	Present in one	–	Japanese	Tsukada et al., 2015
p.C542Y	vWFA2	Yes	2nd−5th	Not present	Frequent (82%)	Chinese	Yuan et al., 2008
p.F121S	LCCL	Yes	2nd−3rd	Present in all	Common	USA	Hildebrand et al., 2010
p.F527C	vWFA2	Yes	N.A.	Not present	–	Korean	Cho et al., 2012
p.G38D	LCCL	Yes	N.A.	N.A.	N.A.	Korean	Choi et al., 2013
p.G87V	LCCL	Yes	4th	Present in all	Sometimes	Chinese	Chen et al., 2013
p.G87W	LCCL	Yes	5th	Present in all	–	Dutch	Collin et al., 2006; Pauw et al., 2007a,b
p.G88E	LCCL	Yes	4th−7th	Present in some	–	Dutch, USA	Kemperman et al., 2005
p.I109N	LCCL	Yes	2nd−3rd	Present in all	–	Australian	Kamarinos et al., 2001
p.I109T	LCCL	Yes	4th−6th	Present in all	–	Dutch	Pauw et al., 2007b
p.I372T	vWFA2	Yes	4th−5th	Not present	–	Japanese	Tsukada et al., 2015
p.Ile399_Ala404del	vWFA2	Yes	3rd	Not present	Common	USA	Gallant et al., 2013
p.L114P	LCCL	Yes	N.A.	N.A.	N.A.	Korean	Choi et al., 2013; Burgess et al., 2016
p.M512T	vWFA2	Yes	5th	Not present	Sometimes	Chinese	Yuan et al., 2008
p.P51S	LCCL	Yes	2rd−5th	Present in all	Common	Dutch, USA	Lemaire et al., 2003; Bischoff et al., 2005
p.P89H	LCCL	Yes	Congenital	N.A.	–	USA	Dodson et al., 2012
p.V104del	LCCL	Yes	4th	Present in all	–	Hungarian	Nagy et al., 2004
p.V123E	LCCL	Yes	4th−6th	Not present	–	N.A.	Jung et al., 2015
p.V66G	LCCL	Yes	2nd−3rd	Present in some	–	USA	Grabski et al., 2003
p.W117R	LCCL	Yes	3rd	Present in some	–	Korean, USA	Nagy et al., 2008; Tsukada et al., 2015

*Decade of onset of either hearing loss, vestibular symptoms or both. In most mutations hearing loss precedes vestibular symptoms by a few years; N.A., no data available; Dutch, The Netherlands and Belgium*.

Several COCH mutations originate from specific locations such as North America, Japan, Australia, Korea, China, and Belgium/the Netherlands. For the Pro51Ser mutation a Dutch founder was discovered (de Kok et al., 1999; Verhagen et al., 2001).

### Pathophysiology

The DFNA9 mutations lead to the production of mutated cochlin. Since the exact physiological role of cochlin is yet to be discovered, the pathophysiological relevance of mutated cochlin is still unknown.

Different pathophysiological mechanisms have been described for different mutations, although they lead to similar phenotypes (Liepinsh et al., 2001; Robertson et al., 2003; Yao et al., 2010; Bae et al., 2014; Jung et al., 2015). For the mutations p.V104del, p.I109T, and p.F121S located on the Limulus factor C, cochlin, and LCCL domain and p.C162Y, p.A487P located on the vWFA domains Bae et al. found that they were not secreted into the media (Yao et al., 2010). A failure in transport from the endoplasmatic reticulum to the Golgi complex leading to accumulation within the cell was documented for the latter mutations. Dimeric aggregates and multimeric aggregates are the product of protein misfolding (Grabski et al., 2003; Robertson et al., 2003; Street et al., 2005; Yao et al., 2010; Cho et al., 2012). LCCL domain mutations were found to form intracellular dimeric aggregates while mutations in vWFA domains lead to the formation of multimeric aggregates. High-molecular-weight aggregates are linked to early onset of hearing loss in DFNA9 patients whereas dimeric aggregates are associated with a later age of onset (Cho et al., 2012; Bae et al., 2014). For the vWFA domain mutations p.F527C and p.C542Y intracellular accumulation of multimeric cochlin was found, however monomeric secretions of mutant cochlin reach similar levels as in wild-type. COCH mutations in the LCCL domain are dominated by vestibular symptoms, while mutations in the vWFA predominantly lead to hearing loss. (Nagy et al., 2004; Bae et al., 2014).

Another pathophysiological mechanism involving decreased secretion is reduced cleavage of intracellular cochlin by aggrecanase, described for p.P51S, p.V66G, p.G88E, p.I109T, p.W117R, p.V123E, and p.C162Y. Wild type cochlin is cleaved by aggrecanase after which the LCCL-domain is secreted into the extracellular compartment. The c-terminal domain remains intracellular (Py et al., 2013; Jung et al., 2015). Because of the misfolding, post-translational cleavage of mutated cochlin is impaired. This leads to a reduction in secretion of the LCCL domain to the extracellular compartment. The absence of cleaved LCCL within the inner ear would lead to a reduced inflammatory reaction because of the innate immune function of LCCL, which eventually leads to an accumulation of endotoxins within the inner ear and the characteristic late-onset bilateral cochleovestibular deficiency (Bae et al., 2014).

Other hypotheses involving the normal secretion of mutated cochlin speak of impaired integration in the extracellular matrix (ECM) leading to structural changes in the matrix of the cochleovestibular system (Grabski et al., 2003; Robertson et al., 2006) or extracellular dimerization and multimerization, this is yet to be further elucidated (Yao et al., 2010; Jung et al., 2015).

### Histology

McCall et al. (2011) found that temporal bones of DFNA9 patients had abnormal mixed eosinophilic deposits within the cochlea and vestibular labyrinth, the tympanic membrane, incudomallear, and incudostapedial joint. In the middle ear, deposits in the tympanic membrane cause a thickening of the middle layer. The deposits resembled the morphology of cartilage. Other mobile areas within the middle ear such as the stapediovestibular joint, tensor tympani tendon, anterior malleal ligament, and incudal ligament did not reliably demonstrate similar deposits. Important to note is that these histological findings are different to those of the inner ear.

Within the inner ear, the presence of eosinophilic cochlin deposits is considered pathognomonic for DFNA9. These deposits are acellular and homogenous as found by Robertson et al. (Goel et al., 2012) Electron microscopy of the inner ear section in DFNA9 patients shows a highly branched, densely packed and haphazardly branched microfibrillar substance. Only one type of microfibrils is detected which is 20 nm or less in size. These findings are in contrast with the extracellular matrix of healthy individuals where multiple types of microfibrils are periodically banded with a moderate amount of space between the bundles (Bom et al., 2003).

### Imaging

Limited evidence is available on sclerotic lesions and/or narrowing of one of the semicircular canals in DFNA9. These findings have been observed on T2-weighted MRI images as a phenotypic radiological feature of DFNA9 by de Varebeke et al. (2014) who also found that the fibrotic process within the inner ear is only later followed by ossification. However, Van Rompaey et al. (2016) demonstrated afterwards that these findings are also present in patients with simultaneous cochleovestibular deficiency compared to patients who only have bilateral deafness. Since there is a correlation between MRI abnormalities and lateral semicircular canal function loss they proposed T2-weighted MRI as possible biomarker for BVL in general.

### Quality of life

BVL often leads to a decreased QoL, as reported by Guinand et al. (2012), who have studied QoL in a general BVL population, including four DFNA9 patients. Several health-related QoL instruments were used to study the impact of BVL overall, including the Short-Form Health Survey (SF-36), Dizziness Handicap Inventory (DHI), and the Short Falls Efficacy Scale-International (Short FES-I) (Guinand et al., 2012). Physical functioning, general health, vitality, and social functioning were significantly impaired (compared to the general Dutch population), meaning that both physical, and mental QoL is significantly decreased in these patients (Guinand et al., 2012). Thirty-three BVL patients (85%) had a score of 30 or higher (moderate self-perceived handicap), of which 17 (44%) had a score of 60 or higher (severe self-perceived handicap). The Short FES-I is a questionnaire used to assess the fear of falling. Only seven patients reported to have no fear of falling, whereas 29 patients (74%) had a slight to moderate fear. Three patients with BVL (8%) had a severe fear of falling. To evaluate oscillopsia, a specific questionnaire was reported by Guinand et al. (2012) Due to its chronic nature patients tend to report less inconvenience by oscillopsia over time due to habituation, though the questionnaire still revealed a significant impact on daily life.

Vermeire et al. (Vermeire et al., 2006; Castiglione et al., 2016) describe a significant improvement in QoL and speech-recognition in DFNA9 patients after cochlear implantation. They used the Hearing Handicap Inventory for Adults, the Glasgow Benefit Inventory, and the Scale for the Prediction of Hearing Disability in SNHL (Vermeire et al., 2006).

### Cognitive impairment

Numerous studies have already described the correlation between hearing impairment and cognitive decline. (Fulton et al., 2015; Miller et al., 2015; Castiglione et al., 2016; Claes et al., 2016) In DFNA9 patients, the impact on QoL of SNHL and cochlear implantation was studied, without focusing on cognitive impairment (Vermeire et al., 2006; Castiglione et al., 2016). No data were available on the evaluation of cognition in DFNA9 patients or BVL in general at the time of the literature search. Afterwards, Popp et al. reported on data suggesting cognitive impairment in BVL patients (Popp et al., 2017).

## Discussion

DFNA9 is an autosomal dominant non-syndromic disorder characterized by late-onset SNHL associated with progressive BVL (Chen et al., 2013). This systematic review is characterized by a transparent search strategy and study selection.

The pathophysiology is not yet fully elucidated, although all documented mutations share roughly the same phenotype, including late-onset SNHL and BVH. Some exceptions have been documented, p.P89H is associated with congenital SNHL, while p.C162Y, p.C542Y, p.F527C, p.I372T, p.Ile399_Ala404del, and p.M512T are not associated with BVL.

QoL has not been studied extensively in DFNA9, although scarce work is available on the positive QoL impact of cochlear implantation to rehabilitate hearing (Vermeire et al., 2006; Castiglione et al., 2016). Currently, no treatments are available clinically to restore vestibular function. The treatment consists mainly of specific exercises and informing the patient. Practical information such as fall risk precautions is discussed (Furman et al., 2010). To restore vestibular function, several centers have studied the possibility of a vestibular implant (Lewis, 2016). In analogy to the cochlear implant, the vestibular implant sends electronic signals to the vestibular system. In doing so, it imitates normal vestibular function and provides the central nervous system with motion information (van de Berg et al., 2011, 2012; Merfeld and Lewis, 2012; Thompson et al., 2012; Guinand et al., 2015). Even in an etiologically heterogeneous patient population (including DFNA9 patients), a vestibular implant could be an effective way to activate the vestibular system and have a significant impact on QoL of BVL patients (Guinand et al., 2015).

Several studies have been conducted on the impact of hearing loss on cognition (Lin et al., 2013; Fulton et al., 2015; Miller et al., 2015; Castiglione et al., 2016; Claes et al., 2016). Lin et al. performed a large prospective study with 1,984 older adults of which there were 1,162 hearing impaired. They evaluated the cognitive state during multiple years, and concluded that hearing loss is an independent factor that causes an acceleration of cognitive decline. The progression of the cognitive decline is linear to the progression of the hearing deterioration. The mechanism of this link between hearing and cognitive decline is still to be further examined (Miller et al., 2015).

Few studies have been conducted on the impact of BVL on cognition. Some animal models and patient studies documented hippocampal deterioration following vestibular dysfunction leading to impaired spatial memory. Also, over several patient studies deficits in memory, spatial memory, perception, attention, and ability to analyze information were found. Moreover, in some cases a correlation was found between the severity of the vestibular symptoms and cognitive impairment (Smith et al., 2005). However, in 2003 an epidemiological study, involving 200 patients, conducted by Gizzi et al. found no significant correlation between the diagnosis of vestibular dysfunction and the presence of cognitive complaints (Gizzi et al., 2003). The correlation between BVL and cognitive tasks involving spatial tasks has always been the strongest. Popp et al. recently found a significant impairment of visuospatial abilities, processing speed, short-term memory. and executive functions in BVL patients. They compared unilateral vestibular loss (UVL) with BVL and controls and found a significant difference in cognitive deterioration between patients with BVL and UVL. For patients with UVL they documented significant impairment in visuospatial skills and reduced processing speed, the latter only in some cases.

Since there is a correlation between BVL and cognitive deterioration specifically in spatial domains, even for non-spatial skills a strong similar deterioration can be expected in DFNA9 patients (Popp et al., 2017). However, DFNA9 patients also suffer from hearing loss, which may also affect immediate memory, language, attention, and delayed memory. We would therefore anticipate a combined detrimental effect on cognition. For this reason, we conducted a systematic review on the available literature. However, we were unable to find studies evaluating cognition in patients affected by BVL and SNHL such as in DFNA9.

There are many instruments to evaluate the cognitive function of a patient. The most frequently used are the Montreal Cognitive Assessment (MoCa), Mini-Mental State Exam (MMSE), Addenbrooke's Cognitive Examination revised version (ACE-R), Repeatable Battery for the Assessment of Neuropsychological Status (RBANS), Neurobehavioral Cognitive Screening Examination (NCSE), etc. When comparing MMSE, MoCa, and RBANS a strong correlation is found between the score of RBANS and MOCA, while only RBANS has a strong correlation with total brain volume (Paul et al., 2011; Lin et al., 2017). While the MOCA can screen for mild cognitive impairment and the MMSE is used more frequently in Alzheimer's disease, the RBANS has more potential as a tool in case repeated measures are required, e.g. prospective follow-up studies. The RBANS also has the benefit of generating one outcome measure (instead of focussing on one aspect such as d2, Boston Name, etc.) and its easy application in an ENT clinic. Two other tools that also offer this benefit are Alzheimer's Disease Assessment Scale Cognitive (ADAS-Cog) and Mattis Dementia Rating Scale (MDRS), however ADAS-Cog does not test executive functions, which was found to be impaired in patients with vestibular dysfunction. (Popp et al., 2017) Both MDRS and RBANS require verbal communication with the patient, which makes the results questionable when used in studies including patients with hearing impairment (Appels and Scherder, 2010). However, an adjusted version of the RBANS, the RBANS-H, has been drafted especially for patients with impaired hearing, overcoming the bias caused by hearing impairment within these cognitive assessment tools (Claes et al., 2016).

## Conclusion

Cognitive impairment has been demonstrated in several studies in patients with hearing loss and in one study on patients with bilateral vestibular failure. However, no studies have been reported in DFNA9, where patients are affected by combined otovestibular failure. Further research is warranted to correlate otovestibular status with cognition.

## Author contributions

JD, SM, AC, GM, PVdH, and VV conceived and designed the study. JD, SM, and VV drafted the manuscript and have undertaken data collection and analysis. AC and GM critically revised the manuscript. All authors read and approved the final manuscript. All authors agree to be accountable for all aspects of the work in ensuring that questions related to the accuracy or integrity of any part of the work are appropriately investigated and resolved.

### Conflict of interest statement

The authors declare that the research was conducted in the absence of any commercial or financial relationships that could be construed as a potential conflict of interest.

## References

[B1] Appels B. A. Scherder E. (2010). The diagnostic accuracy of dementia-screening instruments with an administration time of 10 to 45 minutes for use in secondary care: a systematic review. Am. J. Alzheimers. Dis. Other Demen. 25, 301–316. 10.1177/1533317510367485 20539025 PMC10845578

[B2] Bae S. H. Robertson N. G. Cho H. J. Morton C. C. Jung D. J. Baek J. I.. (2014). Identification of pathogenic mechanisms of COCH mutations, abolished cochlin secretion, and intracellular aggregate formation: genotype–phenotype correlations in DFNA9 deafness and vestibular disorder. Hum. Mut. 35, 1506–1513. 10.1002/humu.22701 25230692 PMC4373469

[B3] Besnard S. Lopez C. Brandt T. Denise P. Smith P. F. (2015). Editorial: the vestibular system in cognitive and memory processes in mammalians. Front. Integr. Neurosci. 9:55. 10.3389/fnint.2015.00055 26617498 PMC4639622

[B4] Bessot N. Denise P. Toupet M. Van Nechel C. Chavoix C. (2012). Interference between walking and a cognitive task is increased in patients with bilateral vestibular loss. Gait Posture 36, 319–321. 10.1016/j.gaitpost.2012.02.021 22465706

[B5] Bischoff A. M. Huygen P. L. Kemperman M. H. Pennings R. J. Bom S. J. Verhagen W. I.. (2005). Vestibular deterioration precedes hearing deterioration in the P51S COCH mutation (DFNA9): an analysis in 74 mutation carriers. Otol. Neurotol. 26, 918–925. 10.1097/01.mao.0000185048.84641.e3 16151338

[B6] Bischoff A. M. Pauw R. J. Huygen P. L. Aandekerk A. L. Kremer H. Cremers C. W.. (2007). Vertical corneal striae in families with autosomal dominant hearing loss: DFNA9/COCH. Am. J. Ophthalmol. 143:847.e6–852.e6. 10.1016/j.ajo.2007.01.037 17368553

[B7] Bom S. J. Kemperman M. H. De Kok Y. J. Huygen P. L. Verhagen W. I. Cremers F. P.. (1999a). Progressive cochleovestibular impairment caused by a point mutation in the COCH gene at DFNA9. Laryngoscope 109, 1525–1530. 10.1097/00005537-199909000-00031 10499067

[B8] Bom S. J. Kemperman M. H. Huygen P. L. Luijendijk M. W. Cremers C. W. (2003). Cross-sectional analysis of hearing threshold in relation to age in a large family with cochleovestibular impairment thoroughly genotyped for DFNA9/COCH. Ann. Otol. Rhinol. Laryngol. 112, 280–286. 10.1177/000348940311200316 12656423

[B9] Bom S. J. Kunst H. P. Huygen P. L. Cremers F. P. Cremers C. W. (1999b). Non-syndromal autosomal dominant hearing impairment: ongoing phenotypical characterization of genotypes. Br. J. Audiol. 33, 335–348. 10.3109/03005369909090117 10890148

[B10] Brandt T. Schautzer F. Hamilton D. A. Brüning R. Markowitsch H. J. Kalla R.. (2005). Vestibular loss causes hippocampal atrophy and impaired spatial memory in humans. Brain 128, 2732–2741. 10.1093/brain/awh617 16141283

[B11] Burgess B. J. O'Malley J. T. Kamakura T. Kristiansen K. Robertson N. G. Morton C. C.. (2016). Histopathology of the human inner ear in the p.L114P COCH mutation (DFNA9). Audiol. Neurootol. 21, 88–97. 10.1159/000443822 27023102 PMC4833584

[B12] Castiglione A. Benatti A. Velardita C. Favaro D. Padoan E. Severi D.. (2016). Aging, cognitive decline and hearing loss: effects of auditory rehabilitation and training with hearing aids and cochlear implants on cognitive function and depression among older adults. Audiol. Neurootol. 21(Suppl. 1), 21–28. 10.1159/000448350 27806352

[B13] Chen D. Y. Chai Y. C. Yang T. Wu H. (2013). Clinical characterization of a novel COCH mutation G87V in a Chinese DFNA9 family. Int. J. Pediatr. Otorhinolaryngol. 77, 1711–1715. 10.1016/j.ijporl.2013.07.031 23993205

[B14] Cho H. J. Park H. J. Trexler M. Venselaar H. Lee K. Y. Robertson N. G.. (2012). A novel COCH mutation associated with autosomal dominant nonsyndromic hearing loss disrupts the structural stability of the vWFA2 domain. J. Mol. Med. 90, 1321–1331. 10.1007/s00109-012-0911-2 22610276 PMC4361775

[B15] Choi B. Y. Park G. Gim J. Kim A. R. Kim B. J. Kim H. S.. (2013). Diagnostic application of targeted resequencing for familial nonsyndromic hearing loss. PLoS ONE 8:e68692. 10.1371/journal.pone.0068692 23990876 PMC3750053

[B16] Claes A. J. Mertens G. Gilles A. Hofkens-Van den Brandt A. Fransen E. Van Rompaey V.. (2016). The repeatable battery for the assessment of neuropsychological status for hearing impaired individuals (RBANS-H) before and after cochlear implantation: a protocol for a prospective, longitudinal cohort study. Front. Neurosci. 10:512. 10.3389/fnins.2016.00512 27895549 PMC5108794

[B17] Collin R. W. Pauw R. J. Schoots J. Huygen P. L. Hoefsloot L. H. Cremers C. W. (2006). Identification of a novel COCH mutation, G87W, causing autosomal dominant hearing impairment (DFNA9). Am. J. Med. Genet. Part A 140A, 1791–1794. 10.1002/ajmg.a.31354 16835921

[B18] de Kok Y. J. Bom S. J. Brunt T. M. Kemperman M. H. van Beusekom E. van der Velde-Visser S. D.. (1999). A Pro51Ser mutation in the COCH gene is associated with late onset autosomal dominant progressive sensorineural hearing loss with vestibular defects. Hum. Mol. Genet. 8, 361–366. 10.1093/hmg/8.2.361 9931344

[B19] de Varebeke S. P. Termote B. Van Camp G. Govaerts P. J. Schepers S. Cox T.. (2014). Focal sclerosis of semicircular canals with severe DFNA9 hearing impairment caused by a P51S COCH-mutation: is there a link? Otol. Neurotol. 35, 1077–1086. 10.1097/MAO.0000000000000283 24662630

[B20] Dodson K. M. Georgolios A. Barr N. Nguyen B. Sismanis A. Arnos K. S.. (2012). Etiology of unilateral hearing loss in a national hereditary deafness repository. Am. J. Otolaryngol. 33, 590–594. 10.1016/j.amjoto.2012.03.005 22534022

[B21] Fanselow M. S. Dong H. W. (2010). Are the dorsal and ventral hippocampus functionally distinct structures? Neuron 65, 7–19. 10.1016/j.neuron.2009.11.031 20152109 PMC2822727

[B22] Fransen E. Verstreken M. Verhagen W. I. Wuyts F. L. Huygen P. L. D'Haese P.. (1999). High prevalence of symptoms of menière's disease in three families with a mutation in the COCH gene. Hum. Mol. Genet. 8, 1425–1429. 10.1093/hmg/8.8.1425 10400989

[B23] Fulton S. E. Lister J. J. Bush A. L. Edwards J. D. Andel R. (2015). Mechanisms of the hearing-cognition relationship. Sem. Hear. 36, 140–149. 10.1055/s-0035-1555117 27516714 PMC4906307

[B24] Furman J. M. Raz Y. Whitney S. L. (2010). Geriatric vestibulopathy assessment and management. Curr. Opin. Otolaryngol. Head Neck Surg. 18, 386–391. 10.1097/MOO.0b013e32833ce5a6 20613528 PMC4879828

[B25] Gallacher J. Ilubaera V. Ben-Shlomo Y. Bayer A. Fish M. Babisch W.. (2012). Auditory threshold, phonologic demand, and incident dementia. Neurology 79, 1583–1590. 10.1212/WNL.0b013e31826e263d 23019269

[B26] Gallant E. Francey L. Fetting H. Kaur M. Hakonarson H. Clark D.. (2013). Novel COCH mutation in a family with autosomal dominant late onset sensorineural hearing impairment and tinnitus. Am. J. Otolaryngol. 34, 230–235. 10.1016/j.amjoto.2012.11.002 23374487

[B27] Gao J. Xue J. Chen L. Ke X. Qi Y. Liu Y. (2013). Whole exome sequencing identifies a novel DFNA9 mutation, C162Y. Clin. Genet. 83, 477–481. 10.1111/cge.12006 22931125

[B28] Gizzi M. Zlotnick M. Cicerone K. Riley E. (2003). Vestibular disease and cognitive dysfunction: no evidence for a causal connection. J. Head Trauma Rehabil. 18, 398–407. 10.1097/00001199-200309000-00002 12973270

[B29] Goel M. Sienkiewicz A. E. Picciani R. Wang J. Lee R. K. Bhattacharya S. K. (2012). Cochlin, intraocular pressure regulation and mechanosensing. PLoS ONE 7:e34309. 10.1371/journal.pone.0034309 22496787 PMC3319572

[B30] Göttlich M. Jandl N. M. Sprenger A. Wojak J. F. Münte T. F. Krämer U. M.. (2016). Hippocampal gray matter volume in bilateral vestibular failure. Hum. Brain Mapp. 37, 1998–2006. 10.1002/hbm.23152 26918638 PMC6867258

[B31] Grabski R. Szul T. Sasaki T. Timpl R. Mayne R. Hicks B.. (2003). Mutations in COCH that result in non-syndromic autosomal dominant deafness (DFNA9) affect matrix deposition of cochlin. Hum. Genet. 113, 406–416. 10.1007/s00439-003-0992-7 12928864

[B32] Green S. H. J. (2014). Cochrane Handbook for Systematic Reviews of Interventions Version 5.0.2. Wiley-Blackwell (Accessed September 1, 2009).

[B33] Guinand N. Boselie F. Guyot J. P. Kingma H. (2012). Quality of life of patients with bilateral vestibulopathy. Ann. Otol. Rhinol. Laryngol. 121, 471–477. 10.1177/000348941212100708 22844867

[B34] Guinand N. van de Berg R. Ranieri M. Cavuscens S. DiGiovanna J. Nguyen T. A. (2015). Vestibular implants: hope for improving the quality of life of patients with bilateral vestibular loss, in Conference proceedings : Annual International Conference of the IEEE Engineering in Medicine and Biology Society IEEE Engineering in Medicine and Biology Society Conference 2015 (Milan), 7192–7195. 10.1109/EMBC.2015.7320051 26737951

[B35] Gurgel R. K. Ward P. D. Schwartz S. Norton M. C. Foster N. L. Tschanz J. T. (2014). Relationship of hearing loss and dementia: a prospective, population-based study. Otol. Neurotol. 35, 775–781. 10.1097/MAO.0000000000000313 24662628 PMC4024067

[B36] Hildebrand M. S. Gandolfo L. Shearer A. E. Webster J. A. Jensen M. Kimberling W. J.. (2010). A novel mutation in COCH—implications for genotype-phenotype correlations in DFNA9 hearing loss. Laryngoscope 120, 2489–2493. 10.1002/lary.21159 21046548 PMC3329724

[B37] Jung J. Kim H. S. Lee M. G. Yang E. J. Choi J. Y. (2015). Novel COCH p.V123E mutation, causative of DFNA9 sensorineural hearing loss and vestibular disorder, shows impaired cochlin post-translational cleavage and secretion. Hum. Mut. 36, 1168–1175. 10.1002/humu.22855 26256111

[B38] Kamarinos M. McGill J. Lynch M. Dahl H. (2001). Identification of a novel COCH mutation, I109N, highlights the similar clinical features observed in DFNA9 families. Hum. Mut. 17:351. 10.1002/humu.37 11295836

[B39] Kemperman M. H. De Leenheer E. M. Huygen P. L. van Duijnhoven G. Morton C. C. Robertson N. G.. (2005). Audiometric, vestibular, and genetic aspects of a DFNA9 family with a G88E COCH mutation. Otol. Neurotol. 26, 926–933. 10.1097/01.mao.0000185062.12458.87 16151339

[B40] Khetarpal U. (2000). DFNA9 is a progressive audiovestibular dysfunction with a microfibrillar deposit in the inner ear. Laryngoscope 110, 1379–1384. 10.1097/00005537-200008000-00030 10942145

[B41] Kremmyda O. Hüfner K. Flanagin V. L. Hamilton D. A. Linn J. Strupp M.. (2016). Beyond dizziness: virtual navigation, spatial anxiety and hippocampal volume in bilateral vestibulopathy. Front. Hum. Neurosci. 10:139. 10.3389/fnhum.2016.00139 27065838 PMC4814552

[B42] Lemaire F. X. Feenstra L. Huygen P. L. Fransen E. Devriendt K. Van Camp G.. (2003). Progressive late-onset sensorineural hearing loss and vestibular impairment with vertigo (DFNA9/COCH): longitudinal analyses in a belgian family. Otol. Neurotol. 24, 743–748. 10.1097/00129492-200309000-00009 14501450

[B43] Lewis R. F. (2016). Vestibular implants studied in animal models: clinical and scientific implications. J. Neurophysiol. 116, 2777–2788. 10.1152/jn.00601.2016 27760820 PMC5148793

[B44] Li L. Ikezono T. Watanabe A. Shindo S. Pawankar R. Yagi T. (2005). Expression of full-length Cochlin p63s is inner ear specific. Auris Nasus Larynx 32, 219–223. 10.1016/j.anl.2005.03.012 15885953

[B45] Liepinsh E. Trexler M. Kaikkonen A. Weigelt J. Bányai L. Patthy L.. (2001). NMR structure of the LCCL domain and implications for DFNA9 deafness disorder. EMBO J. 20, 5347–5353. 10.1093/emboj/20.19.5347 11574466 PMC125649

[B46] Lin F. R. Metter E. J. O'Brien R. J. Resnick S. M. Zonderman A. B. Ferrucci L. (2011). Hearing loss and incident dementia. Arch. Neurol. 68, 214–220. 10.1001/archneurol.2010.362 21320988 PMC3277836

[B47] Lin F. R. Yaffe K. Xia J. Xue Q. L. Harris T. B. Purchase-Helzner E.. (2013). Hearing loss and cognitive decline in older adults. JAMA Intern. Med. 173, 293–299. 10.1001/jamainternmed.2013.1868 23337978 PMC3869227

[B48] Lin V. Y. Chung J. Callahan B. L. Smith L. Gritters N. Chen J. M.. (2017). Development of cognitive screening test for the severely hearing impaired: hearing-impaired MoCA. Laryngoscope 127(Suppl. 1), S4–S11. 10.1002/lary.26590 28409842

[B49] Lundin-Olsson L. Nyberg L. Gustafson Y. (1997). “Stops walking when talking” as a predictor of falls in elderly people. Lancet 349:617. 10.1016/S0140-6736(97)24009-2 9057736

[B50] Manolis E. N. Yandavi N. Nadol J. B. Eavey R. D. McKenna M. Rosenbaum S.. (1996). A gene for non-syndromic autosomal dominant progressive postlingual sensorineural hearing loss maps to chromosome 14q12–13. Hum. Mol. Genet. 5, 1047–1050. 10.1093/hmg/5.7.1047 8817345

[B51] Martini A. Castiglione A. Bovo R. Vallesi A. Gabelli C. (2014). Aging, cognitive load, dementia and hearing loss. Audiol. Neurootol. 19(Suppl. 1), 2–5. 10.1159/000371593 25733358

[B52] McCall A. A. Linthicum F. H. O'Malley J. T. Adams J. C. Merchant S. N. Bassim M. K.. (2011). Extralabyrinthine manifestations of DFNA9. J. Assoc. Res. Otolaryngol. 12, 141–149. 10.1007/s10162-010-0245-0 21052762 PMC3046331

[B53] Merfeld D. M. Lewis R. F. (2012). Replacing semicircular canal function with a vestibular implant. Curr. Opin. Otolaryngol. Head Neck Surg. 20, 386–392. 10.1097/MOO.0b013e328357630f 22886037

[B54] Miller G. Miller C. Marrone N. Howe C. Fain M. Jacob A. (2015). The impact of cochlear implantation on cognition in older adults: a systematic review of clinical evidence. BMC Geriatr. 15:16. 10.1186/s12877-015-0014-3 25879461 PMC4348398

[B55] Nagy I. Horváth M. Trexler M. Répássy G. Patthy L. (2004). A novel COCH mutation, V104del, impairs folding of the LCCL domain of cochlin and causes progressive hearing loss. J. Med. Genet. 41:e9. 10.1136/jmg.2003.012286 14729849 PMC1757273

[B56] Nagy I. Trexler M. Patthy L. (2008). The second von willebrand type A domain of cochlin has high affinity for type I, type II and type IV collagens. FEBS Lett. 582, 4003–4007. 10.1016/j.febslet.2008.10.050 19013156

[B57] Paul R. Lane E. M. Tate D. F. Heaps J. Romo D. M. Akbudak E.. (2011). Neuroimaging signatures and cognitive correlates of the montreal cognitive assessment screen in a nonclinical elderly sample. Arch. Clin. Neuropsychol. 26, 454–460. 10.1093/arclin/acr017 21642663 PMC3142949

[B58] Pauw R. J. Collin R. W. Huygen P. L. Hoefsloot L. H. Kremer H. Cremers C. W. (2007a). Clinical characteristics of a Dutch DFNA9 family with a novel COCH mutation, G87W. Audiol. Neurootol. 12, 77–84. 10.1159/000097794 17264471

[B59] Pauw R. J. Huygen P. L. Colditz G. M. Cremers C. W. (2011). Phenotype analysis of an Australian DFNA9 family with the 1109N COCH mutation. Ann. Otol. Rhinol. Laryngol. 120, 414–421. 10.1177/000348941112000612 21774451

[B60] Pauw R. J. Huygen P. L. Collin R. W. Cruysberg J. R. Hoefsloot L. H. Kremer H.. (2007b). Phenotype description of a novel DFNA9/COCH mutation, I109T. Ann. Otol. Rhinol. Laryngol. 116, 349–357. 10.1177/000348940711600506 17561763

[B61] Peracino A. (2014). Hearing loss and dementia in the aging population. Audiol. Neurootol. 19(Suppl. 1), 6–9. 10.1159/000371595 25733359

[B62] Popp P. Wulff M. Finke K. Rühl M. Brandt T. Dieterich M. (2017). Cognitive deficits in patients with a chronic vestibular failure. J. Neurol. 264, 554–563. 10.1007/s00415-016-8386-7 28074268

[B63] Py B. F. Gonzalez S. F. Long K. Kim M. S. Kim Y. A. Zhu H.. (2013). Cochlin produced by follicular dendritic cells promotes antibacterial innate immunity. Immunity 38, 1063–1072. 10.1016/j.immuni.2013.01.015 23684986 PMC3758559

[B64] Robertson N. G. Cremers C. W. Huygen P. L. Ikezono T. Krastins B. Kremer H.. (2006). Cochlin immunostaining of inner ear pathologic deposits and proteomic analysis in DFNA9 deafness and vestibular dysfunction. Hum. Mol. Genet. 15, 1071–1085. 10.1093/hmg/ddl022 16481359

[B65] Robertson N. G. Hamaker S. A. Patriub V. Aster J. C. Morton C. C. (2003). Subcellular localisation, secretion, and post-translational processing of normal cochlin, and of mutants causing the sensorineural deafness and vestibular disorder, DFNA9. J. Med. Genet. 40, 479–486. 10.1136/jmg.40.7.479 12843317 PMC1735525

[B66] Robertson N. G. Jones S. M. Sivakumaran T. A. Giersch A. B. Jurado S. A. Call L. M.. (2008). A targeted Coch missense mutation: a knock-in mouse model for DFNA9 late-onset hearing loss and vestibular dysfunction. Hum. Mol. Genet. 17, 3426–3434. 10.1093/hmg/ddn236 18697796 PMC2566528

[B67] Robertson N. G. Resendes B. L. Lin J. S. Lee C. Aster J. C. Adams J. C.. (2001). Inner ear localization of mRNA and protein products of COCH, mutated in the sensorineural deafness and vestibular disorder, DFNA9. Hum. Mol. Genet. 10, 2493–2500. 10.1093/hmg/10.22.2493 11709536

[B68] Scholten R. J. P. M. Offringa M. Assendelft W. J. J. (2014). Inleiding in Evidence-Based Medicine. Houten: Bohn Stafleu van Loghum.

[B69] Smith P. F. Zheng Y. Horii A. Darlington C. L. (2005). Does vestibular damage cause cognitive dysfunction in humans? J. Vest. Res. 15, 1–9. 15908735

[B70] Street V. A. Kallman J. C. Robertson N. G. Kuo S. F. Morton C. C. Phillips J. O. (2005). A novel DFNA9 mutation in the vWFA2 domain of COCH alters a conserved cysteine residue and intrachain disulfide bond formation resulting in progressive hearing loss and site-specific vestibular and central oculomotor dysfunction. Am. J. Med. Genet. A 139A, 86–95. 10.1002/ajmg.a.30980 16261627

[B71] Stroup D. F. Berlin J. A. Morton S. C. Olkin I. Williamson G. D. Rennie D.. (2000). Meta-analysis of observational studies in epidemiology: a proposal for reporting. Meta-analysis of observational studies in epidemiology (MOOSE) group. JAMA 283, 2008–2012. 10.1001/jama.283.15.2008 10789670

[B72] Thompson L. A. Haburcakova C. Gong W. Lee D. J. Wall C. III. Merfeld D. M.. (2012). Responses evoked by a vestibular implant providing chronic stimulation. J. Vest. Res. 22, 11–15. 10.3233/VES-2012-0442 22699148 PMC4041130

[B73] Tsukada K. Ichinose A. Miyagawa M. Mori K. Hattori M. Nishio S. Y.. (2015). Detailed hearing and vestibular profiles in the patients with COCH mutations. Ann. Otol. Rhinol. Laryngol. 124(Suppl. 1):100S−110S. 10.1177/0003489415573074 25780252

[B74] van de Berg R. Guinand N. Guyot J. P. Kingma H. Stokroos R. J. (2012). The modified ampullar approach for vestibular implant surgery: feasibility and its first application in a human with a long-term vestibular loss. Front. Neurol. 3:18. 10.3389/fneur.2012.00018 22363317 PMC3282298

[B75] van de Berg R. Guinand N. Stokroos R. J. Guyot J. P. Kingma H. (2011). The vestibular implant: quo vadis? Front. Neurol. 2:47. 10.3389/fneur.2011.00047 21991260 PMC3181464

[B76] Van Rompaey V. De Belder F. Parizel P. Van de Heyning P. (2016). Semicircular canal fibrosis as a biomarker for lateral semicircular canal function loss. Front. Neurol. 7:43. 10.3389/fneur.2016.00043 27047448 PMC4803745

[B77] Verbecque E. Marijnissen T. De Belder N. Van Rompaey V. Boudewyns A. Van de Heyning P.. (2017). Vestibular (dys)function in children with sensorineural hearing loss: a systematic review. Int. J. Audiol. 56, 361–381. 10.1080/14992027.2017.1281444 28264605

[B78] Verhagen W. I. Bom S. J. Fransen E. Van Camp G. Huygen P. L. Theunissen E. J.. (2001). Hereditary cochleovestibular dysfunction due to a COCH gene mutation (DFNA9): a follow-up study of a family. Clin. Otolaryngol. Allied Sci. 26, 477–483. 10.1046/j.1365-2273.2001.00505.x 11843927

[B79] Vermeire K. Brokx J. P. Wuyts F. L. Cochet E. Hofkens A. De Bodt M.. (2006). Good speech recognition and quality-of-life scores after cochlear implantation in patients with DFNA9. Otol. Neurotol. 27, 44–49. 10.1097/01.mao.0000187240.33712.01 16371846

[B80] Wuyts F. L. Van Rompaey V. Maes L. K. (2016). “SO STONED”: common sense approach of the dizzy patient. Front. Surg. 3:32. 10.3389/fsurg.2016.00032 27313999 PMC4887462

[B81] Yao J. Py B. F. Zhu H. Bao J. Yuan J. (2010). Role of protein misfolding in DFNA9 hearing loss. J. Biol. Chem. 285, 14909–14919. 10.1074/jbc.M110.106724 20228067 PMC2865277

[B82] Yuan H. J. Han D. Y. Sun Q. Yan D. Sun H. J. Tao R.. (2008). Novel mutations in the vWFA2 domain of COCH in two Chinese DFNA9 families. Clin. Genet. 73, 391–394. 10.1111/j.1399-0004.2008.00972.x 18312449

